# Viral Infections of the Oral Cavity in Children

**DOI:** 10.3390/children10081325

**Published:** 2023-07-31

**Authors:** Alessandra Amato

**Affiliations:** Department of Neuroscience, Reproductive Science and Dentistry, University of Naples Federico II, 80131 Naples, Italy; aale.amato@gmail.com

Various viral infections can affect the oral cavities of pediatric patients [1,2]. Some viral infections are more severe in children than they are in adults, and vice versa [1,2]. Children and adults respond differently to viral infections, as the maturity of specific immunity influences the clinical course of the disease [2]. Indeed, the specific immunity of neonates and children is inherently immature or can be modulated by tolerance induction in ways that impair it [1].

Viral infections of the oral cavity can be distinguished into those that do not result in visible damage or a disease in the oral cavity, but are transmitted orally or during dental procedures, and those associated with oral and perioral lesions [2,3]. However, some of them belong to both categories [2,4].

Primary infection with herpes simplex virus-1 is generally acquired during early childhood and is usually subclinical or underdiagnosed [5]. The primary infection manifests in the characteristic condition known as herpetic gingivostomatitis, which is characterized by painful, small vesicles bilaterally covering the gingival and oral mucosa [5]. Recurrent herpes simplex virus-1 infection is called herpes labialis or cold sores. It occurs unilaterally because the virus reactivates and latently infects some neurons in the trigeminal ganglion [6,7]. This is often triggered by a co-infection, sun exposure, or stress [7,8]. The symptomatic period can be shortened by the local administration of a paste containing aciclovir at the site of prodromal symptoms or immediately at the first sign of a blister [2]. There is no cure for herpes simplex virus-1, which can cause rare complications, such as facial paralysis, which is called Bell’s palsy [9]. However, primary herpes simplex virus-1 infection can be treated with aciclovir, and recurrences can be treated with topical pastes containing aciclovir [2,5].

Human papillomaviruses (HPV) can cause benign and malignant diseases in various areas, such as the genital and oral mucosa or skin [10]. Infections with specific HPV genotypes have been associated with an increased risk of cervical cancer (HPV-16 and -18) and head and neck cancer (HPV-16) [11]. Vertical transmission via mothers is the most common route of HPV transmission in children younger than one year [2]. In children younger than 18 years, the most common HPV-related oral manifestations are, in descending order, focal epithelial hyperplasia, squamous cell papillomas, verrucae vulgaris, and condylomata acuminata [10]. Although the oncogenic role of HPV in oral squamous cell carcinoma in children is still unclear [10], vaccination is recommended at a younger age regardless of the patient’s HPV status to improve its effectiveness [12].

SARS-CoV-2 infections occurred less frequently among children than they do among adults during the early phase of the pandemic, possibly because infected children were asymptomatic or had only mild symptoms [1]. However, some children with COVID-19 were later diagnosed with Kawasaki disease (KD), “Multisystem Inflammatory Syndrome in Children” (MIS-C), and other syndromes [1]. They are more likely to develop ulcerative/erosive, macular-petechial, especially erythematous oral mucosa lesions [1] than adults are, who are usually diagnosed with white plaques and erosive/ulcerative, maculopapular, or vesicular lesions [13]. COVID-19 vaccinations have been effective in protecting against the infection, even in pediatric subjects [14,15], and have been associated mainly with mild adverse effects, including local pain, swelling, and redness at the injection site, general weakness, joint or muscle pain, headache, chills, fever, nausea, and diarrhea [16]. A few adverse effects have been reported in children, including oral lesions with erosive-ulcerative phenotype, which is similar to adults [1]. In contrast, white lesions, such as lichenoid reactions and oral lichen planus, have been described only in adults [9].

Enteroviruses are RNA viruses that are usually fecally–orally transmitted [17]. They belong to the Picornaviridae family, have a single-stranded genome of about 9000 bases, and are acid-stable, allowing them to survive low pH in the stomach [17,18]. Among them, coxsackie A virus is the most common cause of hand, foot, and mouth disease, which is characterized by fever and blisters in the mouth and extremities [17]. However, Enteroviruses can also cause meningoencephalitis or severe pulmonary disease in children [17], and no antivirals are currently available [17].

Morbilli virus is a negative-stranded RNA virus that belongs to the paramyxovirus family. It is transmitted via direct contact and has an incubation period of less than one week [19]. Morbilli virus causes measles, which are characterized by a flu-like upper respiratory illness with a fever, followed by the onset of a red, blotchy exanthema that covers most of the body [19,20]. Measles can lead to severe complications such as subsclerosing panencephalitis, which can cause permanent brain damage [19,21]. The MMR vaccine is highly effective against measles, mumps, and rubella, and its use is strongly recommended [22]. It is also worth considering that the association between the MMR vaccination and autism has not been confirmed [22].

Mumps virus infection typically leads to inflammation and the swelling of the parotid gland, resulting in parotitis [23,24]. The infection heals spontaneously within 1–2 weeks, resulting in lifelong protection [23,24]. Infection with the mumps virus can lead to male infertility, again underlining the importance of the MMR vaccination [2].

Human immunodeficiency virus type 1 is most commonly transmitted vertically in children from an infected mother during childbirth, in utero, or through breastfeeding [25]; although, antiviral therapy administered to the mother before the child is born can effectively reduce the risk of transmission [26]. HIV-1 infection suppresses the host T cell response, leading to opportunistic infections similar to those seen in transplant patients or individuals with general immunosuppression [2]. Candida albicans causes most opportunistic infections in pediatric patients diagnosed with acquired immunodeficiency syndrome (AIDS) [2,27,28]. Effective antifungal therapies are available to treat Candida infections [29] that, combined with effective antiretroviral therapy, favor immune system recovery and opportunistic infection resolution [28]. However, there is a risk of developing a refractory HIV infection if the prescribed drug therapy is not strictly adhered to [2,30]. Combination therapies for viral infections have pioneered the treatment of HIV-1 infections, with at least three antiviral agents currently being used in therapy [26]. While the first HIV-1 inhibitor was developed in 1985, the therapy was revolutionized in 1995 with the introduction of protease inhibitors [26]. Future drugs for HIV infection will have a longer half-life and reduce the need for strict adherence to the drug therapy [31]. Existing preparations are also being reformulated to include multiple drugs in a single tablet and preparations with a longer half-life.

Vaccination programs have effectively prevented severe childhood infections, although viral infections remain a serious health problem in developing countries [14,25]. Emerging infections, such as human immunodeficiency virus (HIV), further contribute to the burden of viral infections during childhood [28]. Antiviral therapies will likely play a more significant role in treating childhood infections [5]. As new infections emerge, vaccines or antiviral agents promise to prevent or treat these infections and change the landscape of viral infections among children [5].

## Data Availability

Data are available on Google Scholar, Web of Science, Pubmed, and Scopus databases.

## References

[1] Di Spirito F., Caggiano M., Di Palo M.P., Contaldo M., D’Ambrosio F., Martina S., Amato A. (2022). Oral Lesions in Pediatric Subjects: SARS-CoV-2 Infection and COVID-19 Vaccination. Appl. Sci..

[2] Sällberg M. (2009). Oral Viral Infections of Children. Periodontology 2000.

[3] Di Spirito F. (2022). Oral-Systemic Health and Disorders: Latest Prospects on Oral Antisepsis. Appl. Sci..

[4] Di Spirito F., Argentino S., Martuscelli R., Sbordone L. (2019). MRONJ incidence after multiple teeth extractions in patients taking oral bis-phosphonates without “drug holiday”: A retrospective chart review. Oral Implantol..

[5] Huang C.-W., Hsieh C.-H., Lin M.-R., Huang Y.-C. (2020). Clinical Features of Gingivostomatitis Due to Primary Infection of Herpes Simplex Virus in Children. BMC Infect. Dis..

[6] Steiner I., Kennedy P.G., Pachner A.R. (2007). The Neurotropic Herpes Viruses: Herpes Simplex and Varicella-Zoster. Lancet Neurol..

[7] Hedner E., Vahlne A., Kahnberg K.E., Hirsch J.M. (1993). Reactivated Herpes Simplex Virus Infection as a Possible Cause of Dry Socket after Tooth Extraction. J. Oral. Maxillofac. Surg..

[8] Hedner E., Vahlne A., Hirsch J.-M. (1990). Primary Herpes Simplex Virus (Type 1) Infection Delays Healing of Oral Excisional and Extraction Wounds in the Rat. J. Oral. Pathol. Med..

[9] Di Spirito F., Amato A., Di Palo M.P., Contaldo M., D’Ambrosio F., Lo Giudice R., Amato M. (2022). Oral Lesions Following Anti-SARS-CoV-2 Vaccination: A Systematic Review. Int. J. Environ. Res. Public. Health.

[10] Di Spirito F., Pantaleo G., Di Palo M.P., Amato A., Raimondo A., Amato M. (2023). Oral Human Papillomavirus Benign Lesions and HPV-Related Cancer in Healthy Children: A Systematic Review. Cancers.

[11] Zur Hausen H. (1994). Disrupted Dichotomous Intracellular Control of Human Papillomavirus Infection in Cancer of the Cervix. Lancet.

[12] Paavonen J., Jenkins D., Bosch F.X., Naud P., Salmerón J., Wheeler C.M., Chow S.-N., Apter D.L., Kitchener H.C., Castellsague X. (2007). Efficacy of a Prophylactic Adjuvanted Bivalent L1 Virus-like-Particle Vaccine against Infection with Human Papillomavirus Types 16 and 18 in Young Women: An Interim Analysis of a Phase III Double-Blind, Randomised Controlled Trial. Lancet.

[13] Di Spirito F., Iandolo A., Amato A., Caggiano M., Raimondo A., Lembo S., Martina S. (2022). Prevalence, Features and Degree of Association of Oral Lesions in COVID-19: A Systematic Review of Systematic Reviews. Int. J. Environ. Res. Public. Health.

[14] Di Spirito F., Contaldo M., Amato A., Di Palo M.P., Pantaleo G., Amato M. (2022). COVID-19 Vaccine and Oral Lesions: Putative Pathogenic Mechanisms. Oral. Dis..

[15] Di Spirito F., Pelella S., Argentino S., Sisalli L., Sbordone L. (2022). Oral Manifestations and the Role of the Oral Healthcare Workers in COVID-19. Oral. Dis..

[16] Luzzi V., Ierardo G., Bossù M., Polimeni A. (2021). Paediatric Oral Health during and after the COVID-19 Pandemic. Int. J. Paediatr. Dent..

[17] Bible J.M., Pantelidis P., Chan P.K.S., Tong C.Y.W. (2007). Genetic Evolution of Enterovirus 71: Epidemiological and Pathological Implications. Rev. Med. Virol..

[18] Bonderoff J.M., Lloyd R.E. (2008). CVB Translation: Lessons from the Polioviruses. Group B Coxsackieviruses.

[19] Schneider-Schaulies J., ter Meulen V., Schneider-Schaulies S. (2003). Measles Infection of the Central Nervous System. J. Neurovirol..

[20] Duke T., Mgone C.S. (2003). Measles: Not Just Another Viral Exanthem. Lancet.

[21] Tierney L.M., Wang K.C. (2006). Koplik’s Spots. N. Engl. J. Med..

[22] Smeeth L., Cook C., Fombonne E., Heavey L., Rodrigues L.C., Smith P.G., Hall A.J. (2004). MMR Vaccination and Pervasive Developmental Disorders: A Case-Control Study. Lancet.

[23] Hviid A., Rubin S., Mühlemann K. (2008). Mumps. Lancet.

[24] Su S.-B., Chang H.-L., Chen K.-T. (2020). Current Status of Mumps Virus Infection: Epidemiology, Pathogenesis, and Vaccine. Int. J. Env. Environ. Res. Public. Health.

[25] Prendergast A., Tudor-Williams G., Jeena P., Burchett S., Goulder P. (2007). International Perspectives, Progress, and Future Challenges of Paediatric HIV Infection. Lancet.

[26] MacArthur R.D., Novak R.M., Peng G., Chen L., Xiang Y., Hullsiek K.H., Kozal M.J., van den Berg-Wolf M., Henely C., Schmetter B. (2006). A Comparison of Three Highly Active Antiretroviral Treatment Strategies Consisting of Non-Nucleoside Reverse Transcriptase Inhibitors, Protease Inhibitors, or Both in the Presence of Nucleoside Reverse Transcriptase Inhibitors as Initial Therapy (CPCRA 058 FIRST Study): A Long-Term Randomised Trial. Lancet.

[27] Boccia G., Di Spirito F., D’Ambrosio F., Di Palo M.P., Giordano F., Amato M. (2023). Local and Systemic Antibiotics in Peri-Implantitis Management: An Umbrella Review. Antibiotics.

[28] Pienaar E.D., Young T., Holmes H. (2010). Interventions for the Prevention and Management of Oropharyngeal Candidiasis Associated with HIV Infection in Adults and Children. Cochrane Database Syst. Rev..

[29] D’Ambrosio F., Di Spirito F., Amato A., Caggiano M., Lo Giudice R., Martina S. (2022). Attitudes towards Antibiotic Prescription and Antimicrobial Resistance Awareness among Italian Dentists: What Are the Milestones?. Healthcare.

[30] D’Ambrosio F., Di Spirito F., De Caro F., Lanza A., Passarella D., Sbordone L. (2022). Adherence to Antibiotic Prescription of Dental Patients: The Other Side of the Antimicrobial Resistance. Healthcare.

[31] Deeks S.G. (2003). Treatment of Antiretroviral-Drug-Resistant HIV-1 Infection. Lancet.

